# Future directions and management of liquid biopsy in non-small cell lung cancer

**DOI:** 10.37349/etat.2020.00015

**Published:** 2020-08-31

**Authors:** Alessia Maria Cossu, Marianna Scrima, Angela Lombardi, Anna Grimaldi, Margherita Russo, Alessandro Ottaiano, Michele Caraglia, Marco Bocchetti

**Affiliations:** 1 Biogem Scarl, Institute of Genetic Research, Laboratory of Molecular and Precision Oncology, 83031 Ariano Irpino, Italy; 2 Department of Precision Medicine, University of Campania “Luigi Vanvitelli”, 80138 Naples, Italy; 3 Department of Abdominal Oncology, SSD-Innovative Therapies for Abdominal Cancers, Istituto Nazionale Tumori di Napoli, IRCCS “G. Pascale”, Via M. Semmola, 80131 Naples, Italy; Università degli studi della Campania, Italy

**Keywords:** Non-small cell lung cancer, biomarkers, liquid biopsy, circulating cell-free tumor DNA, molecular analysis

## Abstract

Lung cancer represents the world’s most common cause of cancer death. In recent years, we moved from a generic therapeutic strategy to a personalized approach, based on the molecular characterization of the tumor. In this view, liquid biopsy is becoming an important tool for assessing the progress or onset of lung disease. Liquid biopsy is a non-invasive procedure able to isolate circulating tumor cells, tumor educated platelets, exosomes and free circulating tumor DNA from body fluids. The characterization of these liquid biomarkers can help to choose the therapeutic strategy for each different case. In this review, the authors will analyze the main aspects of lung cancer and the applications currently in use focusing on the benefits associated with this approach for predicting the prognosis and monitoring the clinical conditions of lung cancer disease.

## Introduction

**Figure 1.**
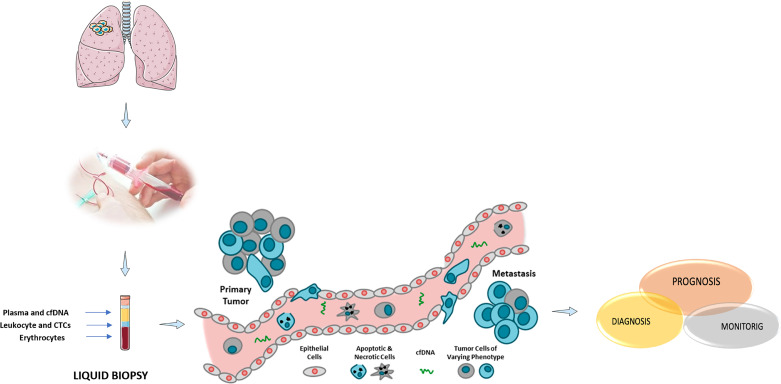
Overview of molecular liquid biopsy components. cfDNA: cell-free DNA

Lung cancer is among the most diagnosed tumor in the world and it represents the major cause of demise. Over 1.2 million lung cancer deaths are registered [1]. Lung cancer is categorized in two main groups: small cell lung cancer (approximately 20%) and non-small cell lung cancer (NSCLC, approximately 80%) [2]. The 5-year survival rate of lung cancer patients is between 4% and 17%, according to different stages and regions [3]. Cigarette smoking represents the main risk factor. However, the risk of carcinoma development can be hereditary and probably derives from the presence of a rare autosomal dominant gene frequent in the population [4]. The high mortality rate is often due to late diagnosis, given when the tumor is already at an advanced stage and most patients will not respond to therapy. Previous disclosures underlined a precious method for the screening of asymptomatic NSCLC patients in a potentially treatable stage, in order to improve the healing. Lung cancer diagnosis is made by radiography, sputum cytology, bronchoscopy, needle biopsy, and other techniques. Adjuvant chemotherapy combined with radiotherapy can produce a survival advantage compared to the adjuvant irradiation. In cases of advanced and extensive NSCLC combination chemotherapy is usually recommended, in particular with drugs such as paclitaxel, docetaxel, and gemcitabine, etc. Surgery has only a limited role in the management of NSCLC. The combination of platinum-based chemotherapy plus radiation therapy is required for patients with locally advanced disease [5]. Currently, mutations in the epidermal growth factor receptor (*EGFR*) and translocations involving the anaplastic lymphoma kinase (*ALK*) gene are among the most important targets for targeted therapy [6]. In addition, many mutations in the B-Raf proto-oncogene (*BRAF*), human epidermal growth factor receptor 2 (*HER2*), hepatocyte growth factor receptor coding gene (*MET*), phosphatidylinositol-4,5-bisphosphate 3-kinase catalytic subunit alpha (*PIK3CA*), ROS proto-oncogene 1 (*ROS1*), proto-oncogene tyrosine-protein kinase receptor coding gene (*RET*), protein kinase B (*AKT*), discoidin domainreceptor tyrosine kinase 2 (*DDR2*), and K-Ras protein coding gene (*KRAS*) genes have a certain relevance for target therapy [7]. In recent years, several new strategies targeting *EGFR* mutations, *ALK* fusions, *ROS1* fusions, *BRAF* V600E mutations, and neurotrophin receptor tyrosine kinase (*NTRK*) fusions are emerging [8]. Moreover, some immune checkpoint inhibitors such as T lymphocyte-associated cytotoxic protein 4 (CTLA-4) and programmed death-1 (PD-1) and its ligands (PD-L1 and PD-L2) have been proposed to escape the immune system surveillance, opening the way for the evolution of specific monoclonal antibodies [9]. The use of conventional chemotherapy, oncogene target agents, immune system, or T cell checkpoint inhibitors showed significantly higher efficacy against early-stage tumors. Therefore, some more specific and sensible diagnostic tests are intertwined with new therapies development: the more tumor detection in the early initial phase is efficient; the better is the response to the therapy. A valid approach in high-risk subjects is represented by low dose computed tomography (LDCT). LDCT helps in the early diagnosis of the tumor, together with the traditional tissue biopsy to confirm the characteristics of the nodule observed by the CT scan. In fact, this method detects more than twice the number of lung tumors at an early stage and has been associated with a significant reduction in specific mortality of lung cancer compared to chest X-ray [10]. Despite its high sensitivity, both the National Lung Screening Trial (NLST) and other European studies showed relatively low specificity, with a high false-positive rate [11]. In addition, the NSLT study found that LDTC reduced mortality slightly; this evidence has raised some doubts about the benefit of LDCT in high-risk populations [12, 13]. The diagnosis of metastatic NSCLC is often based on small biopsies or on cytological samples that sometimes are not sufficiently suitable for optimal molecular analysis. Furthermore, tissue biopsy may not necessarily represent the global molecular panorama of the tumor because it is performed on a specific site of a non-heterogeneous tumor. In this light, the use of less invasive techniques has become essential, complementary with radiological approaches currently in use. A valid contribution for prognostic and diagnostic screening is represented by the evaluation of circulating biomarkers. Liquid biopsy compared to tissue biopsy certainly has the advantage of being a non-invasive evaluation technique of tumor-specific biomarkers present in body fluids [14]. In particular, liquid biopsy provides an in-depth knowledge of the genetic profile of cancer and metastases, improving survival times and promoting selective therapy that can increase or suppress cellular growth; on the contrary, tissue biopsy is less effective, because it can only provide limited tumor vision in a single time point [15]. In fact, the disadvantages of tissue biopsy led the oncology field to switch the focus on the evaluation of the circulating components in the bloodstream because they are more effective for monitoring the disease and following any newly acquired resistance to therapy. Liquid biopsy is able to isolate and analyze different components derived from the tumor and provide essential information to characterize its molecular profile and capture metastatic heterogeneity [16]. These components, isolated from the bloodstream, urine, cerebrospinal fluid, saliva and pleural effusions [17], are circulating tumor DNA (ctDNA), cell-free tumor RNA (ctRNA), exosomes, miRNA, tumor-educated platelets (TEPs), and circulating cancer cells (CTCs) (Figure 1).

Liquid biopsy can detect a considerable number of mutations, like point mutations, small insertions and deletions. However, this approach could detect other genetic aberrations such as the size of the DNA fragments, variations in the number of copies, translocations and epigenetic alterations. There are several highly sensitive digital systems for detecting circulating tumor DNA. These technologies such as polymerase chain reaction (PCR), real-time quantitative polymerase chain reaction (qPCR), the digital droplet-polymerase chain reaction (ddPCR), beads, emulsion, amplification and magnetics (BEAMing) are widely used in the cancer research field and only recently, next generation sequencing (NGS) has been shown to be a valid system in the evaluation of tumor specific alterations, allowing the sequencing of small amount of nucleic acids. Personalized medicine is now a crucial approach based on exploiting patients’ genetic information to select or optimize the adjuvant therapy tied to the specific tumor and to individual’s need. ctDNA analysis is essential since it enlightens about tumor heterogeneity and clonal evolution and provides a more complete molecular profile about changing subclone population and increase therapy efficacy. The important information on the gene changes during the disease obtained from the liquid biopsy increase the interest of this approach [18].

Therefore, it can be useful for early diagnosis of relapses before tumors become radiographically or clinically evident, offering to clinicians a wider window opportunity in which the treatment regimens could be modified [19]. Once a patient relapses, liquid biopsies can reveal new mutations not originally present in the primary tumor that could guide a choice for second-line therapy. The approaches identified in liquid biopsy are summarized here in order to point out the importance of these methods in diagnosing, prognosis and monitoring the treatment of disease.

## Aspects of circulating tumor biomarkers in liquid biopsies

### CTCs

**Figure 2.**
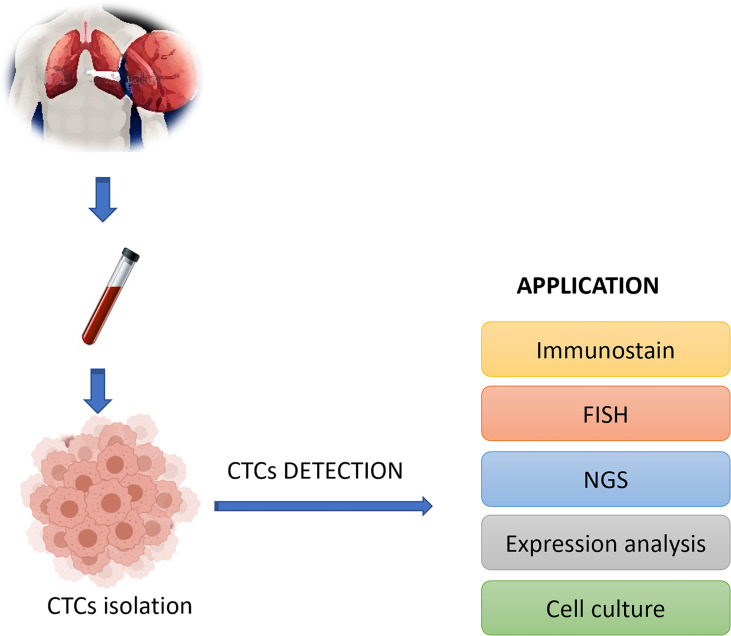
Applications of CTC technologies

Recent studies have highlighted CTCs role as potential prognostic, diagnostic and monitoring biomarkers [20]. CTCs provide a real tumor snapshot (DNA, RNA and proteins) ready to be analyzed and they might prove useful to perform *ex vivo* functional studies and cultures, as well [16]. The isolation of CTCs occurs through methods presenting a great variability in detection rates, sensitivity and specificity. There is only one technique approved by the U.S. Food and Drug Administration (FDA) for CTCs isolation: CellSearch™ (Veridex LLC), i.e. magnetic microspheres with anti-epithelial cell adhesion molecule antibodies (EpCAM). This technique has been approved for metastatic breast, colorectal or prostate cancer [21, 22]. Isolation of CTCs is very demanding in NSCLC, compared to other cancers since it is a tumor with high aggressiveness and invasiveness. In fact, the isolation of CTCs in NSCLC is less effective. CTCs can be lost as they undergo to epithelial-mesenchymal transition (EMT) and for the down-regulate of their epithelial markers during progression. Therefore, the initial EMT does not have the ability to resize the epithelial properties of cancer cells and it is difficult to discriminate those markers with existing techniques [23]. Several techniques can be used in NSCLC metastatic tumors such as the “CTC-chip” characterized by the use of antibodies to isolate CTCs: a technique that successfully detects CTCs in the blood with 50% of purity [24]. Other techniques take advantage of intrinsic properties, such as size (e.g., ISET; Rare Cell Diagnostics, Paris, France), deformability, or dielectric sensitivity and/or negative selection of white blood cells [25, 26]. Some mutations like *EGFR*, *KRAS* or the expression of *MET* have been detected in lung cancer. Their analysis becomes an additional method to complement the ctDNA test to facilitate monitoring of the patients response for a personalized therapy [27–30]. In a recent work, a technique called ISET was used to detect malignant circulating cells in patients with asymptomatic NSCLC [31]. However, several studies have enlightened the relevance of CTCs as prognostic and diagnostic sources of information. CTCs have been used as valid biomarkers to prevent lung tumors, as showed in chronic obstructive pulmonary disease [32]. Similarly, a study on early-stage NSCLC patients suggested that folate receptor positive CTCs could be used as diagnostic biomarkers [33]. However, despite the positive results regarding the value of CTCs as diagnostic biomarkers, there is no reliable evidence in the clinic in NSCLC [34]. In fact, new technologies to detect CTCs are now being developed (Figure 2). The methods currently in use are represented by erythrocyte lysis and cell centrifugation with long processing times and high costs. For these reasons, it is necessary to develop a system capable of limiting the loss of CTCs during isolation and increase the level of purity and establish a standard protocol to efficiently isolate vital CTCs suitable for *in vitro* expansion and *ex vivo* clinical applications [35]. A valid alternative to those systems is represented by nanotechnologies, with unique physical properties able to overcome the limits of traditional CTC detection methods [36].

### cfDNA and ctDNA

ctDNA is a fraction of cfDNA which differs from normal circulating DNA and it is characterized by specific mutations in oncogenes or suppressor genes [14, 16]. Consequently, ctDNA is more concentrated in sick than in healthy subjects [37]. Cancer specific ctDNA provides molecular clues on the fragmented DNA of tumors and their specific mutations [35]. The ctDNA release mechanisms in the bloodstream are not yet clear, but it has been assumed that it can be released following apoptosis or cell lysis due to necrosis [38]. ctDNA is important because it can provide a picture of what are the mutations occurred to oncogenes or tumor suppressor genes, genetic amplification or epigenetic alterations of the patient [39]. Generally, ctDNA appears fragmented and mixed with non-tumor DNA at very low concentrations (0.01–1% cfDNA), thus making it difficult to quantify it in the early stage of the disease.

It is possible to discriminate ctDNA from cfDNA through ultra-sensitive analytical assays. The methods currently in use to analyze the extracted cfDNA are PCR, digital PCR (dPCR), ddPCR, the clamp-based peptide acid-based PCR test, peptide nucleic acid (PNA) Taqman dosage, microspheres, BEAMing, polymerization managed by pyrophosphorolysis and new NGS [40]. The NGS technique advanced technology can detect multiple mutations present through a single test in a very short time [41]. Sozzi et al. [17], showed a sensitive and specific real-time qPCR test, efficient for plasma DNA evaluation; it represents a new non-invasive approach to identify individuals with lung cancer or with the risk to develop the disease. Gautschi et al. [42], demonstrated that the plasma and serum DNA concentration was higher in NSCLS subjects than in healthy subjects.

It is clear that the quantification of cfDNA could be exploited as an additional screening together with those currently in use including chest CT scans and cytological/histological examination, because it is more promising and effective in the prognosis and diagnosis of carcinoma. Currently, in addition to the NGS technique, Cancer Personal Profiling through deep Sequencing (CAPP-Seq) also represents a valid tool; it is an even more sensitive approach, capable of eliminating wild-type background DNA from normal cells and detecting multiple classes of somatic mutations. Therefore, it appears useful to monitor treatment response, residual disease and progression before radiological approaches [43]. Although cfDNA shows an important prognostic and diagnostic value, clinical application is still limited due to the lack of standardization.

### Exosomes and miRNAs

Exosomes are extracellular vesicles measuring between 30–100 nm. Almost all cell subtypes are able to secrete those endocytosis generated vesicles, that’s why it is possible to find them in different body fluids like pleural and cerebrospinal fluid, saliva, sperm and of course plasma and serum [44]. Exosomes contain different cellular product such as proteins, DNA, mRNAs, and miRNAs. Exosomes are crucial intermediaries between cells in a variety of diseases, including cancer, but also in physiological circumstances [45]. These vesicles are involved in tumor growth, progression, drug resistance and in the preparation of metastatic niche [16, 46]. Different techniques are described to help with their isolation. It is possible to extract exosomes from the above-mentioned fluids by density centrifugation or ultracentrifugation, transmission microscopy, and protein markers exploitation (CD9, CD63, tetraspanin proteins). Extracellular vesicular matrix or immunobead precipitation [16, 47] are also described as isolation methods. Exosomes with tumor origin can contain oncogenic material, causing cancerous transformations in receiving cells. An example is the transfer of EGFR to vascular endothelial cells, together with their own vascular endothelial growth factor (VEGF) [48]. The extraction of miRNAs from biological fluids could give some information about somatic mutations, junction variants, or valuable knowledge on protein and gene expression using enzyme-linked immunosorbent assay (ELISA) or Western Blot analysis together with NGS and real time polymerase chain reaction (RT-PCR) profiling. miRNAs contained inside exosomes are physically protected from RNAse degradation and their concentration in the bloodstream is generally higher. miRNAs are studied extensively due to their potential use diagnostic biomarkers in pulmonary adenocarcinoma [49]. Under this light, the most promising exosomal miRNAs are miR-21, miR-146, miR-17-3p, miR-210, miR-106a, miR-203, miR-214, miR-155, miR-212, miR-191, and miR-192. miR-23a, in particular, is involved in lung cancer progression by suppressing the development of propylhydroxylase, increasing the accumulation of hypoxia-inducible factor 1-α, facilitating the transendothelial passage of malignant cells [50]. miR-151a-5p, miR-30a-3p, miR-100, miR-200b-5p, miR154 and miR-629 were also highlighted as diagnostic biomarkers important to discriminate pulmonary granuloma and adenocarcinoma [51]. A recent study using miRNA-seq showed a unique expression pattern shared between squamous cell carcinoma (SCC) and stage I adenocarcinoma patients. In particular, an exosomal prophylactic profile of tumor-derived miRNA was analyzed showing 80.65% and 83.33% sensitivity and 91.67% and 90.32% specificity for adenocarcinoma and SCC diagnosis, suggesting their potential role in NSCLC early diagnosis as non-invasive and high sensitive biomarker [52]. The exosomes in lung cancer serum patients also mediate EMT and metastasis in healthy cells by increasing vimentin expression [53]. Moreover, exosomes surface membrane proteins like CD317, CD91 and EGFR might represent potential tumor makers [54]. miRNAs are believed important in tumor profiling and crucial for early diagnosis, treatment response, and cancer recurrence marker and may predict the overall survival, as well.

### TEPs

After red cells, platelets are the most abundant cells within the bloodstream. Platelets can be considered immune system “scanning soldiers”, sensing the presence of bacteria, cross-communicating with lymphocytes, and partially regulating immune cell extravasation [55]. They have a significant role in several pathological processes like EMT, which increases the cancer cells ability to penetrate and invade distant tissues exploiting the circulation; they also create a neovascularization supportive environment by stimulating the expression of proangiogenic factor secretion such as platelet-derived growth factor (PDGF), VEGF, and basic fibroblast growth factor (bFGF) [56, 57], inducing tumor cell apoptosis and anoikis and epithelial-mesenchymal switch in tumor cells by direct physical interaction and release of transforming growth factor beta (TGFβ) molecules [58]. Platelets act protecting circulating tumor cells from the normal immune response, through several growth and proangiogenic factors release [59] significantly contributing to metastatization [60]. They are highly involved in tumor microenvironment and important factors in cancer biology as they contribute to tumorigenesis and progression, and to response to therapy. Moreover, platelets form a cell–fibrin–platelet-aggregate surrounding CTCs providing mechanical protection [61]. TEPs can be considered non-invasive biomarkers suitable for RNAs panels determination. In fact, spliced TEP RNA surrogate signatures might give specific information about the presence, localization and cancer molecular phenotype. Platelets carry high amount of genetic material: RNAs, such as miRNAs, pre-mRNAs, mRNAs, circular RNAs (cirRNA), long noncoding RNAs, and mitochondrial DNA [62]. Certainly, platelet activation can induce subsequent pre-mRNA splicing and protein translation [63]. Platelets count and size give information about the putative tumor presence; in fact, a high count is associated to increased mortality in different tumors such as malignant mesothelioma, gynaecological malignancies and lung, kidney, gastric, colon cancers rectum and breast [64]. In NSCLC, tumor-derived platelet factor 4 [PF4, chemokine (C-X-C motif ) ligand 4 (CXCL4)] [65] has been reported to promote megacarbonite-mediated platelet production in the bone marrow and an RNA panel was altered in TEPs in metastatic NSCLC patients [66]. Spliced RNA profiles are easily identifiable by minimal amounts of platelet RNA (100–500 picograms) through the thromboSeq platform, a methodology based on RNA sequencing. This allowed the metastatic carcinoma patients individuation with accuracy between 84% and 96% and identified surrogate signatures of spliced RNAs, associated with the molecular subtype of the tumor tissue, such as *EGFR* and *KRAS* mutations and *HER2* and *MET* amplification [67]. An algorithm, called swarm-intelligence, has recently been implemented and iteratively optimizes the RNA panel to select a set of biomarkers [68]. This system allows the diagnosis of advanced stage NSCLC with an accuracy of 89% in an independent validation cohort. Nilsson et al. [69], through RT-PCR noticed echinoderm microtubule-associated protein-like 4 *(EML4)-ALK* rearrangements in platelets derived from NSCLC patients blood with 100% specificity and 65% sensitivity. *EML4-ALK*+ platelets patients had a remarkably lower progression-free survival: 3.8 months, compared with 16 months showed by patients with *EML4-ALK*-platelets. Moreover, 30 months monitoring of EML4-ALK rearrangements in patients platelets revealed the occurrence of crizotinib resistance onset two months earlier than radiologic disease progression [69]. Sheng et al. [70], carried out a support vector machine network analysis on RNA-seq data derived from 402 NSCLC patient platelets, compared with 231 healthy donors. Target genes were 48, and several modules appeared to play crucial roles in this tumor onset and progression, like Wiskott-Aldrich Syndrome Protein Family Member 1 (*WASF1*), Arginine and Serine Rich Coiled-Coil 1 (*RSRC1*), Protein Kinase AMP-Activated Non-Catalytic Subunit Beta 2 (*PRKAB2*), Pyruvate Dehydrogenase E1 Subunit Beta (*PDHB*), Myosin Light Chain 9 (*MYL9*), Tropomyosin 2 (*TPM2*), and Protein Phosphatase 1 Regulatory Subunit 12C (PPP1R12C). In a recent study, Integrin Subunit Alpha 2b (*ITGA2B*), platelet and megakaryocytes transmembrane glycoprotein, involved in platelet aggregation [71] and in thrombasthenia, was identified as NSCLC platelet RNA marker in a test and an independent validation cohort [72]. TEPs RNA analysis may be complementary to currently in use non-invasive screenings to enhance the early stage cancer detection, characterization and monitoring in order to make therapy more efficient.

## Other oncogenic drivers in lung cancer

Other molecular aberrations, named oncogene-addicted, drive both cancer growth and persistence, dramatically changing NSCLC therapy approach. *EGFR* mutations, *ALK* translocations, and *ROS1* rearrangement require to be identified in cancerous biopsy in order to establish the proper therapeutic scheme. For example, *BRAF* V600 mutations identification lead the therapy choice to effective trametinib/dabrafenib combination, while *ROS1* fusions are efficiently treated with crizotinib. Advanced NSCLC can be treated with several EGFR tyrosine kinase inhibitors (TKIs) (erlotinib, afatinib, osimertinib and gefitinib) and ALKi (ceritinib, alectinib and crizotinib) approved both in US and Europe [73]. There are other evidence showing that *BRAF*, *MET*, *ROS1*, *AKT*, *HER2*, *PIK3CA*, *RET*, *KRAS*, and *DDR2* alteration may lead oncogenic transformation in NSCLC [7]. Recently, BRAF and ROS1 targeted therapies were approved for NSCLC in advanced stages [8, 74]. Moreover, novel EGFR and ALK TKIs are being used in therapy since they are effective on refractory tumor resistant to previous generations ones [75]. *EGFR* mutations were discovered in 2004 in NSCLC, when the results of clinical studies with EGFR TKIs resulted comparable to chemotherapy [76, 77]. Second and third generation EGFR-TKIs drugs have been developed and used, respectively afatinib and osimertinib in patients who developed the *EGFR-T790M* mutation that confers resistance to erlotinib and gefitinib [78]. In 2007 an aberrant fusion of EML4 and ALK was discovered in NSCLC patients [79]. Crizotinib, an oral TKI effective against ALK, ROS1 and MET, represented the first therapeutic approach used against this alteration [80]. This drug presented significant advantages compared to older drugs frequently used as first and the second line therapy, so it became the first choice in untreated NSCLC patients showing this alteration. *ROS1* gene mutations were discovered in 2007 in NSCLC with a frequency of 1–2% [81]. Several mutations conferring resistance were reported (*S1986Y/F*, *L1951R*, *D2033N*, *L2026M*), but the most frequent is *G2032R* mutation responsible for steric hindrance preventing the drug to bind correctly to its binding site and exploiting its activity [82]. Ceritinib showed similar activity on *L2026M* resistance mutation compared to brigatinib resistance mutation, but not on *D2033N*, *L1951R* and *G2032R* [83]. Only cabozantinib is active against G2032R mutation but it has severe side effects and toxicity limiting its use [84]. Targeted therapy is a strongly and rapidly growing field, many new alternatives are expected in the upcoming years.

## Conclusion and perspectives

In this report, we briefly highlighted how liquid lung biopsy can be a promising valid tool for the monitoring and early diagnosis of lung cancer, providing important genomic information viable for individualized treatment strategies. We showed the various biomarkers like CTCs, TEPs, ctDNA and exosomes and the relative technologies today in use are important to obtain crucial information about somatic mutations and gene alterations leading to tumor progression and development. Moreover, miRNAs profiling could be useful to couple with a standard radiological screening test in order to improve the diagnosis. The analytes used for liquid biopsy have their own advantages and disadvantages that must be accounted when choosing a specific marker answering to a specific clinical question. CTCs are relatively rare in early cancers, but they provide a particularly powerful approach to detect a variety of cancer-specific abnormalities present in advanced cancers, such as androgen receptor splice variants. In personalized medicine era, liquid NSCLC biopsy represents a valid and alternative non-invasive method compared to tissue biopsy, complementary to other techniques currently used for diagnosis and monitoring. Moreover, tissues genomic profile provides a tumor picture limited to a single time point. Liquid biopsy, unlike usual techniques, embraces the complex heterogeneity, especially in time, and the deep biological basis of tumors. Although the use of different high-throughput analytical platforms may result in reproducibility problem, the present manuscript underlines the requirement of improved standardization and methods analytical validation for liquid biopsy. However, liquid lung biopsy methods can become more standardized and offer maximum sensitivity using commercially available platforms and tests. Advances in technology, particularly the introduction of NGS techniques, could help the liquid biopsy even more viable. Each biomarker (ctDNA, CTCs, exosome, and TEPs) has its own characteristics but all of them provide some crucial information about all kind of cancer genomic alterations like point mutations, translocations, amplifications and even epigenetic alterations. (Table 1).

**Table 1.** Different characteristics of biomarkers in liquid biopsy

**Biomarker**	**Technique**	**Application**
CTC	CTC CellSearch™ (Veridex LLC) utilizes ferroparticles and antibodies directed to epithelial targets (EpCAM)	Provide RNAs, DNA and proteins for cancer diagnosis and profiling; give information about progression and metastasis
ctDNA and cfDNA	PCR, dPCR, ddPCR, gripper-based peptide acid-based PCR test. PNA Taqman dosage, microspheres, BEAMing, NGS	High sensitivity (one of the highest) for detection of early lung cancer; information about the presence of mutated genes correlates to poor prognosis
Exosome	Isolation by physical or biological properties; MACS; immune-mediated isolation; sucrose gradient method; ultracentrifugation; after isolation, PCR used to separate the RNA and proteins	Important in early diagnosis and prognosis; information about tumor’s biologic profile, growth rate, metastatic capacity and drug resistance; potential vehicle for therapies
TEP	Extraction of tumor biomolecules and nucleic acids (RNAs)	Give large amount of genetic material (RNA and DNA); diagnosis of lung cancer; treatment monitoring

Circulating tumor DNA assays detecting sensitizing mutations and resistance to EGFR already entered in the clinical practice and the detection of resistance mutations for rearrangements of tyrosine kinase of the ALK receptor will certainly follow in the routine use soon enough. Liquid biopsy offers genomic cancer cell profile through the non-invasive and cheap blood sampling or other low-cost body fluids, thus representing one of the most interesting and rapidly improving fields in lung cancer.

## Abbreviations

AKT: protein kinase B
ALK: anaplastic lymphoma kinase
BEAMing: beads, emulsion, amplification and magnetics
BRAF: B-Raf proto-oncogene
cfDNA: cell-free DNA
CTCs: circulating cancer cells
ctDNA: circulating tumor DNA
ddPCR: digital droplet-polymerase chain reactio
DDR2: discoidin domainreceptor tyrosine kinase 2
dPCR: digital PCR
EGFR: epidermal growth factor receptor
EML4: echinoderm microtubule-associated protein-like 4
EMT: epithelial-mesenchymal transition
EpCAM: epithelial cell adhesion molecule
HER2: human epidermal growth factor receptor 2
KRAS: K-Ras protein coding gene
LDCT: low dose computed tomography
MET: hepatocyte growth factorreceptor coding gene
NGS: next generation sequencing
NLST: National Lung Screening Trial
NSCLC: non-small cell lung cancer
PCR: polymerase chain reaction
PIK3CA: phosphatidylinositol-4,5-bisphosphate 3-kinase catalytic subunit alpha
PNA: peptide nucleic acid
qPCR: quantitative polymerase chain reaction
RET: proto-oncogene tyrosine-protein kinase receptor coding gene
ROS1: ROS proto-oncogene 1
RT-PCR: real time polymerase chain reaction
SCC: squamous cell carcinoma
TEPs: tumor-educated platelets
TKIs: tyrosine kinase inhibitors
VEGF: vascular endothelial growth factor

## Declarations

### Copyright

© The Author(s) 2020.

## References

[1] National Lung Screening Trial Research Team Aberle DR Berg CD Black WC Church TR Fagerstrom RM Galen B The National Lung Screening Trial: overview and study design. Radiology. 2011;258:243–53. 10.1148/radiol.10091808 21045183 PMC3009383

[2] Govindan R Page N Morgensztern D Read W Tierney R Vlahiotis A Changing epidemiology of small-cell lung cancer in the United States over the last 30 years: analysis of the surveillance, epidemiologic, and end results database. J Clin Oncol. 2006;24:4539–44. 10.1200/JCO.2005.04.4859 17008692

[3] Hirsch FR Scagliotti GV Mulshine JL Kwon R Curran WJ Jr Wu YL Lung cancer: current therapies and new targeted treatments. Lancet. 2017;389:299–311. 10.1016/S0140-6736(16)30958-8 27574741

[4] Minna JD Roth JA Gazdar AF. Focus on lung cancer. Cancer Cell. 2002;1:49–52. 10.1016/s1535-6108(02)00027-2 12086887

[5] Cersosimo RJ. Lung cancer: a review. Am J Health Syst Pharm. 2002;59:611–42. 10.1093/ajhp/59.7.611 11944603

[6] Canale M Pasini L Bronte G Delmonte A Cravero P Crinò L Role of liquid biopsy in oncogene-addicted non-small cell lung cancer. Transl Lung Cancer Res. 2019;8 Suppl 3:S265–79. 10.21037/tlcr.2019.09.15 31857950 PMC6894991

[7] Schulze AB Evers G Kerkhoff A Mohr M Schliemann C Berdel WE Future options of molecular-targeted therapy in small cell lung cancer. Cancers (Basel). 2019;11:690. 10.3390/cancers11050690 PMC6562929 31108964

[8] Shaw AT Ou SH Bang YJ Camidge DR Solomon BJ Salgia R Crizotinib in ROS1-rearranged non-small-cell lung cancer. N Engl J Med. 2014;371:1963–71. 10.1056/NEJMoa1406766 25264305 PMC4264527

[9] Bironzo P Di Maio M. A review of guidelines for lung cancer. J Thorac Dis. 2018;10 Suppl 3:S1556–63. 10.21037/jtd.2018.03.54 29951306 PMC5994504

[10] National Lung Screening Trial Research Team Aberle DR Adams AM Berg CD Black WC Clapp JD Fagerstrom RM Reduced lung-cancer mortality with low-dose computed tomographic screening. N Engl J Med. 2011;365:95–409. 10.1056/NEJMoa1102873 PMC4356534 21714641

[11] Patz EF Jr Pinsky P Gatsonis C Sicks JD Kramer BS Tammemägi MC NLST Overdiagnosis Manuscript Writing Team. Overdiagnosis in low-dose computed tomography screening for lung cancer. JAMA Intern Med. 2014;174:269–74. 10.1001/jamainternmed.2013.12738 24322569 PMC4040004

[12] Paci E Puliti D Lopes Pegna A Carrozzi L Picozzi G Falaschi F the ITALUNG Working Group. Mortality, survival and incidence rates in the ITALUNG randomised lung cancer screening trial. Thorax. 2017;72:825–31. 10.1136/thoraxjnl-2016-209825 28377492

[13] Infante M Sestini S Galeone C Marchianò A Lutman FR Angeli E Lung cancer screening with low-dose spiral computed tomography: evidence from a pooled analysis of two Italian randomized trials. Eur J Cancer Prev. 2017;26:324–9. 10.1097/CEJ.0000000000000264 27222939 PMC6861837

[14] Santarpia M Karachaliou N González-Cao M Altavilla G Giovannetti E Rosell R. Feasibility of cell-free circulating tumor DNA testing for lung cancer. Biomark Med. 2016;10:417–30. 10.2217/bmm.16.6 26974841

[15] Perakis S Speicher MR. Emerging concepts in liquid biopsies. BMC Med. 2017;15:75. 10.1186/s12916-017-0840-6 28381299 PMC5382440

[16] Siravegna G Marsoni S Siena S Bardelli A. Integrating liquid biopsies into the management of cancer. Nat Rev Clin Oncol. 2017;14:531–48. 10.1038/nrclinonc.2017.14 28252003

[17] Sozzi G Conte D Leon M Ciricione R Roz L Ratcliffe C Quantification of free circulating DNA as a diagnostic marker in lung cancer. J Clin Oncol. 2003;21:3902–8. 10.1200/JCO.2003.02.006 14507943

[18] Crowley E Di Nicolantonio F Loupakis F Bardelli A. Liquid biopsy: monitoring cancer-genetics in the blood. Nat Rev Clin Oncol. 2013;10:472–84. 10.1038/nrclinonc.2013.110 23836314

[19] Tie J Wang Y Tomasetti C Li L Springer S Kinde I Circulating tumor DNA analysis detects minimal residual disease and predicts recurrence in patients with stage II colon cancer. Sci Transl Med. 2016;8:346ra92. 10.1126/scitranslmed.aaf6219 PMC5346159 27384348

[20] Hong Y Fang F Zhang Q. Circulating tumor cell clusters: what we know and what we expect (Review). Int J Oncol. 2016;49:2206–16. 10.3892/ijo.2016.3747 27779656 PMC5117994

[21] Allard WJ Matera J Miller MC Repollet M Connelly MC Rao C Tumor cells circulate in the peripheral blood of all major carcinomas but not in healthy subjects or patients with nonmalignant diseases. Clin Cancer Res. 2004;10:6897–904. 10.1158/1078-0432.CCR-04-0378 15501967

[22] Cristofanilli M Budd GT Ellis MJ Stopeck A Matera J Miller MC Circulating tumor cells, disease progression, and survival in metastatic breast cancer. N Engl J Med. 2004;351:781–91. 10.1056/NEJMoa040766 15317891

[23] De Craene B Berx G. Regulatory networks defining EMT during cancer initiation and progression. Nat Rev Cancer. 2013;13:97–110. 10.1038/nrc3447 23344542

[24] Nagrath S Sequist LV Maheswaran S Bell DW Irimia D Ulkus L Isolation of rare circulating tumour cells in cancer patients by microchip technology. Nature. 2007;450:1235–9. 10.1038/nature06385 18097410 PMC3090667

[25] Raimondi C Nicolazzo C Gradilone A. Circulating tumor cells isolation: the “post-EpCAM era”. Chin J Cancer Res. 2015;27:461–70. 10.3978/j.issn.1000-9604.2015.06.02 26543332 PMC4626820

[26] Desitter I Guerrouahen BS Benali-Furet N Wechsler J Jänne PA Kuang Y A new device for rapid isolation by size and characterization of rare circulating tumor cells. Anticancer Res. 2011;31:427–41. 21378321

[27] Babayan A Alawi M Gormley M Müller V Wikman H McMullin RP Comparative study of whole genome amplification and next generation sequencing performance of single cancer cells. Oncotarget. 2016;8:56066–8. 10.18632/oncotarget.10701 28915574 PMC5593545

[28] Hanssen A Wagner J Gorges TM Taenzer A Uzunoglu FG Driemel G Characterization of different CTC subpopulations in non-small cell lung cancer. Sci Rep. 2016;6:28010. 10.1038/srep28010 27302574 PMC4908396

[29] Maheswaran S Sequist LV Nagrath S Ulkus L Brannigan B Collura CV Detection of mutations in EGFR in circulating lung cancer cells. N Engl J Med. 2008;359:66–77. 10.1056/NEJMoa0800668 PMC3551471 18596266

[30] Mayo C Ortega FG Giménez-Capitán A Molina-Vila MA Serrano MJ Viteri S CK-coated magnetic-based beads as a tool to isolate circulating tumor cells (CTCs) in human tumors. Transl Lung Cancer Res. 2013;2:65–71. 10.3978/j.issn.2218-6751.2013.02.06 25806217 PMC4369853

[31] Fiorelli A Accardo M Carelli E Angioletti D Santini M Di Domenico M. Circulating tumor cells in diagnosing lung cancer: clinical and morphologic analysis. Ann Thorac Surg. 2015;99:1899–905. 10.1016/j.athoracsur.2014.11.049 25678504

[32] Ilie M Hofman V Long-Mira E Selva E Vignaud JM Padovani B “Sentinel” circulating tumor cells allow early diagnosis of lung cancer in patients with chronic obstructive pulmonary disease. PLoS One. 2014;9:e111597. 10.1371/journal.pone.0111597 25360587 PMC4216113

[33] Yu Y Chen Z Dong J Wei P Hu R Zhou C Folate receptor-positive circulating tumor cells as a novel diagnostic biomarker in non-small cell lung cancer. Transl Oncol. 2013;6:697–702. 10.1593/tlo.13535 24466372 PMC3890704

[34] Xu Y Liu B Ding F Zhou X Tu P Yu B Circulating tumor cell detection: a direct comparison between negative and unbiased enrichment in lung cancer. Oncol Lett. 2017;13:4882–6. 10.3892/ol.2017.6046 28599490 PMC5453029

[35] Lin M Chen JF Lu YT Zhang Y Song J Hou S Nanostructure embedded microchips for detection, isolation, and characterization of circulating tumor cells. Acc Chem Res. 2014;47:2941–50. 10.1021/ar5001617 25111636 PMC4204926

[36] Ming Y Li Y Xing H Luo M Li Z Chen J Circulating tumor cells: from theory to nanotechnology-based detection. Front Pharmacol. 2017;8:35. 10.3389/fphar.2017.00035 28203204 PMC5285331

[37] Lim M Kim CJ Sunkara V Kim MH Cho YK. Liquid biopsy in lung cancer: clinical applications of circulating biomarkers (CTCs and ctDNA). Micromachines (Basel). 2018;9:100. 10.3390/mi9030100 PMC6187707 30424034

[38] Jahr S Hentze H Englisch S Hardt D Fackelmayer FO Hesch RD DNA fragments in the blood plasma of cancer patients: quantitations and evidence for their origin from apoptotic and necrotic cells. Cancer Res. 2001;61:1659–65. 11245480

[39] Karachaliou N Gonzalez-Cao M Sosa A Berenguer J Bracht JWP Ito M The combination of checkpoint immunotherapy and targeted therapy in cancer. Ann Transl Med. 2017;5:388. 10.21037/atm.2017.06.47 29114546 PMC5653508

[40] Mayo-de-Las-Casas C Garzón Ibáñez M Jordana-Ariza N García-Peláez B Balada-Bel A Villatoro S An update on liquid biopsy analysis for diagnostic and monitoring applications in non-small cell lung cancer. Expert Rev Mol Diagn. 2018;18:35–45. 10.1080/14737159.2018.1407243 29172773

[41] Forshew T Murtaza M Parkinson C Gale D Tsui DW Kaper F Noninvasive identification and monitoring of cancer mutations by targeted deep sequencing of plasma DNA. Sci Transl Med. 2012;4:136ra68. 10.1126/scitranslmed.3003726 22649089

[42] Gautschi O Bigosch C Huegli B Jermann M Marx A Chassé E Circulating deoxyribonucleic Acid as prognostic marker in non-small-cell lung cancer patients undergoing chemotherapy. J Clin Oncol. 2004;22:4157–64. 10.1200/JCO.2004.11.123 15483026

[43] Newman AM Bratman SV To J Wynne JF Eclov NC Modlin LA An ultrasensitive method for quantitating circulating tumor DNA with broad patient coverage. Nat Med. 2014;20:548–54. 10.1038/nm.3519 24705333 PMC4016134

[44] Zheng H Zhan Y Liu S Lu J Luo J Feng J The roles of tumor-derived exosomes in non-small cell lung cancer and their clinical implications. J Exp Clin Cancer Res. 2018;37:226. 10.1186/s13046-018-0901-5 30217217 PMC6137883

[45] Valadi H Ekström K Bossios A Sjöstrand M Lee JJ Lötvall JO. Exosome-mediated transfer of mRNAs and microRNAs is a novel mechanism of genetic exchange between cells. Nat Cell Biol. 2007;9:654–9. 10.1038/ncb1596 17486113

[46] Mathivanan S Ji H Simpson RJ. Exosomes: extracellular organelles important in intercellular communication. J Proteomics. 2010;73:1907–20. 10.1016/j.jprot.2010.06.006 20601276

[47] Rani S O'Brien K Kelleher FC Corcoran C Germano S Radomski MW Isolation of exosomes for subsequent mRNA, MicroRNA, and protein profiling. Methods Mol Biol. 2011;784:181–95. 10.1007/978-1-61779-289-2_13 21898221

[48] Abdouh M Hamam D Gao ZH Arena V Arena M Arena GO. Exosomes isolated from cancer patients' sera transfer malignant traits and confer the same phenotype of primary tumors to oncosuppressor-mutated cells. J Exp Clin Cancer Res. 2017;36:113. 10.1186/s13046-017-0587-0 28854931 PMC5577828

[49] Reclusa P Taverna S Pucci M Durendez E Calabuig S Manca P Exosomes as diagnostic and predictive biomarkers in lung cancer. J Thorac Dis. 2017;9 Suppl 13:S1373–82. 10.21037/jtd.2017.10.67 29184676 PMC5676107

[50] Wu H Esteve E Tremaroli V Khan MT Caesar R Mannerås-Holm L Metformin alters the gut microbiome of individuals with treatment-naive type 2 diabetes, contributing to the therapeutic effects of the drug. Nat Med. 2017;23:850–8. 10.1038/nm.4345 28530702

[51] Cazzoli R Buttitta F Di Nicola M Malatesta S Marchetti A Rom WN microRNAs derived from circulating exosomes as noninvasive biomarkers for screening and diagnosing lung cancer. J Thorac Oncol. 2013;8:1156–62. 10.1097/JTO.0b013e318299ac32 23945385 PMC4123222

[52] Jin X Chen Y Chen H Fei S Chen D Cai X Evaluation of tumor-derived exosomal miRNA as potential diagnostic biomarkers for early-stage non-small cell lung cancer using next-generation sequencing. Clin Cancer Res. 2017;23:5311–9. 10.1158/1078-0432.CCR-17-0577 28606918

[53] Zomer A Maynard C Verweij FJ Kamermans A Schäfer R Beerling E *In vivo* imaging reveals extracellular vesicle-mediated phenocopying of metastatic behavior. Cell. 2015;161:1046–57. 10.1016/j.cell.2015.04.042 26000481 PMC4448148

[54] Yamashita T Honda M Nakamoto Y Baba M Nio K Hara Y Discrete nature of EpCAM+ and CD90+ cancer stem cells in human hepatocellular carcinoma. Hepatology. 2013;57:1484–97. 10.1002/hep.26168 23174907 PMC7180389

[55] Labelle M Begum S Hynes RO. Platelets guide the formation of early metastatic niches. Proc Natl Acad Sci U S A. 2014;111:E3053–61. 10.1073/pnas.1411082111 25024172 PMC4121772

[56] Janowska-Wieczorek A Wysoczynski M Kijowski J Marquez-Curtis L Machalinski B Ratajczak J Microvesicles derived from activated platelets induce metastasis and angiogenesis in lung cancer. Int J Cancer. 2005;113:752–60. 10.1002/ijc.20657 15499615

[57] Haemmerle M Taylor ML Gutschner T Pradeep S Cho MS Sheng J Platelets reduce anoikis and promote metastasis by activating YAP1 signaling. Nat Commun. 2017;8:310. 10.1038/s41467-017-00411-z 28827520 PMC5566477

[58] Labelle M Begum S Hynes RO. Direct signaling between platelets and cancer cells induces an epithelial-mesenchymal-like transition and promotes metastasis. Cancer Cell. 2011;20:576–90. 10.1016/j.ccr.2011.09.009 22094253 PMC3487108

[59] Klement GL Yip TT Cassiola F Kikuchi L Cervi D Podust V Platelets actively sequester angiogenesis regulators. Blood. 2009;113:2835–42. 10.1182/blood-2008-06-159541 19036702 PMC2661866

[60] McAllister SS Weinberg RA. The tumour-induced systemic environment as a critical regulator of cancer progression and metastasis. Nat Cell Biol. 2014;16:717–27. 10.1038/ncb3015 25082194 PMC6220424

[61] Jiang X Wong KHK Khankhel AH Zeinali M Reategui E Phillips MJ Microfluidic isolation of platelet-covered circulating tumor cells. Lab Chip. 2017;17:3498–503. 10.1039/c7lc00654c 28932842 PMC5690580

[62] Alhasan AA Izuogu OG Al-Balool HH Steyn JS Evans A Colzani M Circular RNA enrichment in platelets is a signature of transcriptome degradation. Blood. 2016;127:e1–11. 10.1182/blood-2015-06-649434 26660425 PMC4797142

[63] Rondina MT Schwertz H Harris ES Kraemer BF Campbell RA Mackman N The septic milieu triggers expression of spliced tissue factor mRNA in human platelets. J Thromb Haemost. 2011;9:748–58. 10.1111/j.1538-7836.2011.04208.x 21255247 PMC3071458

[64] Stone RL Nick AM McNeish IA Balkwill F Han HD Bottsford-Miller J Paraneoplastic thrombocytosis in ovarian cancer. N Engl J Med. 2012;366:610–8. 10.1056/NEJMoa1110352 22335738 PMC3296780

[65] Pucci F Rickelt S Newton AP Garris C Nunes E Evavold C PF4 promotes platelet production and lung cancer growth. Cell Rep. 2016;17:1764–72. 10.1016/j.celrep.2016.10.031 27829148 PMC5108525

[66] Calverley DC Phang TL Choudhury QG Gao B Oton AB Weyant MJ Significant downregulation of platelet gene expression in metastatic lung cancer. Clin Transl Sci. 2010;3:227–32. 10.1111/j.1752-8062.2010.00226.x 21500395 PMC3427741

[67] Best MG Sol N Kooi I Tannous J Westerman BA Rustenburg F RNA-Seq of tumor-educated platelets enables blood-based pan-cancer, multiclass, and molecular pathway cancer diagnostics. Cancer Cell. 2015;28:666–76. 10.1016/j.ccell.2015.09.018 26525104 PMC4644263

[68] Best MG Sol N In 't Veld SGJG Vancura A Muller M Niemeijer AN Swarm intelligence-enhanced detection of non-small-cell lung cancer using tumor-educated platelets. Cancer Cell. 2017;32:238–5. 10.1016/j.ccell.2017.07.004 28810146 PMC6381325

[69] Nilsson RJ Karachaliou N Berenguer J Gimenez-Capitan A Schellen P Teixido C Rearranged EML4-ALK fusion transcripts sequester in circulating blood platelets and enable blood-based crizotinib response monitoring in non-small-cell lung cancer. Oncotarget. 2016;7:1066–75. 10.18632/oncotarget.6279 26544515 PMC4808052

[70] Sheng M Dong Z Xie Y. Identification of tumor-educated platelet biomarkers of non-small-cell lung cancer. Onco Targets Ther. 2018;11:8143–51. 10.2147/OTT.S177384 30532555 PMC6241732

[71] Heidenreich R Eisman R Surrey S Delgrosso K Bennett JS Schwartz E Organization of the gene for platelet glycoprotein IIb. Biochemistry. 1990;29:1232–44. 10.1021/bi00457a020 2322558

[72] Xing S Zeng T Xue N He Y Lai YZ Li HL Development and validation of tumor-educated blood platelets integrin alpha 2b (ITGA2B) RNA for diagnosis and prognosis of non-small-cell lung cancer through RNA-seq. Int J Biol Sci. 2019;15:1977–92. 10.7150/ijbs.36284 31523198 PMC6743295

[73] Tsakonas G Ekman S. Oncogene-addicted non-small cell lung cancer and immunotherapy. J Thorac Dis. 2018;10 Suppl 13:S1547–55. 10.21037/jtd.2018.01.82 29951305 PMC5994494

[74] Baik CS Myall NJ Wakelee HA. Targeting *BRAF*-mutant non-small cell lung cancer: from molecular profiling to rationally designed therapy. Oncologist. 2017;22:786–96. 10.1634/theoncologist.2016-0458 28487464 PMC5507646

[75] Novello S Mazières J Oh IJ de Castro J Migliorino MR Helland Å Alectinib *versus* chemotherapy in crizotinib-pretreated anaplastic lymphoma kinase (ALK)-positive non-small-cell lung cancer: results from the phase III ALUR study. Ann Oncol. 2018;29:1409–16. 10.1093/annonc/mdy121 29668860 PMC6005013

[76] Rosell R Carcereny E Gervais R Vergnenegre A Massuti B Felip E Spanish Lung Cancer Group in collaboration with Groupe Français de Pneumo-Cancérologie and Associazione Italiana Oncologia Toracica. Erlotinib *versus* standard chemotherapy as first-line treatment for European patients with advanced EGFR mutation-positive non-small-cell lung cancer (EURTAC): a multicentre, open-label, randomised phase 3 trial. Lancet Oncol. 2012;13:239–46. 10.1016/S1470-2045(11)70393-X 22285168

[77] Mok TS Wu YL Thongprasert S Yang CH Chu DT Saijo N Gefitinib or carboplatin-paclitaxel in pulmonary adenocarcinoma. N Engl J Med. 2009;361:947–57. 10.1056/NEJMoa0810699 19692680

[78] Mok TS Wu YL Ahn MJ Garassino MC Kim HR Ramalingam SS AURA3 Investigators. Osimertinib or platinum-pemetrexed in EGFR T790M-positive lung cancer. N Engl J Med. 2017;376:629–40. 10.1056/NEJMoa1612674 27959700 PMC6762027

[79] Soda M Choi YL Enomoto M Takada S Yamashita Y Ishikawa S Identification of the transforming EML4-ALK fusion gene in non-small-cell lung cancer. Nature. 2007;448:561–6. 10.1038/nature05945 17625570

[80] Katayama R Lovly CM Shaw AT. Therapeutic targeting of anaplastic lymphoma kinase in lung cancer: a paradigm for precision cancer medicine. Clin Cancer Res. 2015;21:2227–35. 10.1158/1078-0432.CCR-14-2791 25979929 PMC4435823

[81] Bergethon K Shaw AT Ou SH Katayama R Lovly CM McDonald NT ROS1 rearrangements define a unique molecular class of lung cancers. J Clin Oncol. 2012;30:863–70. 10.1200/JCO.2011.35.6345 22215748 PMC3295572

[82] Awad MM Katayama R McTigue M Liu W Deng YL Brooun A Acquired resistance to crizotinib from a mutation in CD74-ROS1. N Engl J Med. 2013;368:2395–401. 10.1056/NEJMoa1215530 23724914 PMC3878821

[83] Gainor JF Tseng D Yoda S Dagogo-Jack I Friboulet L Lin JJ Patterns of metastatic spread and mechanisms of resistance to crizotinib in ROS1-positive non-small-cell lung cancer. JCO Precis Oncol. 2017;[Epub ahead of print]. 10.1200/PO.17.00063 PMC5766287 29333528

[84] Drilon A Rekhtman N Arcila M Wang L Ni A Albano M Cabozantinib in patients with advanced RET-rearranged non-small-cell lung cancer: an open-label, single-centre, phase 2, single-arm trial. Lancet Oncol. 2016;17:1653–60. 10.1016/S1470-2045(16)30562-9 27825636 PMC5143197

